# The Influence of Standardized *Valeriana officinalis* Extract on the CYP3A1 Gene Expression by Nuclear Receptors in* In Vivo* Model

**DOI:** 10.1155/2014/819093

**Published:** 2014-09-11

**Authors:** Anna Bogacz, Przemyslaw M. Mrozikiewicz, Monika Karasiewicz, Joanna Bartkowiak-Wieczorek, Marian Majchrzycki, Przemyslaw L. Mikolajczak, Marcin Ozarowski, Edmund Grzeskowiak

**Affiliations:** ^1^Laboratory of Experimental Pharmacogenetics, Department of Clinical Pharmacy and Biopharmacy, Poznan University of Medical Sciences, Marii Magdaleny 14, 61-861 Poznan, Poland; ^2^Department of Quality Control of Medicinal Products and Dietary Supplements, Institute of Natural Fibres and Medicinal Plants, Libelta 27, 61-707 Poznan, Poland; ^3^Department of Rehabilitation, Poznan University of Medical Sciences, 28 Czerwca 1956, 61-545 Poznan, Poland; ^4^Department of Pharmacology, Poznan University of Medical Sciences, Rokietnicka 5a, 60-806 Poznan, Poland; ^5^Department of Pharmacology and Biotechnology, Institute of Natural Fibres and Medicinal Plants, Libelta 27, 61-707 Poznan, Poland; ^6^Department of Pharmaceutical Botany and Plant Biotechnology, Poznan University of Medical Science, Marii Magdaleny 14, 61-861 Poznan, Poland

## Abstract

*Valeriana officinalis* is one of the most popular medicinal plants commonly used as a sedative and sleep aid. It is suggested that its pharmacologically active compounds derived from the root may modulate the CYP3A4 gene expression by activation of pregnane X receptor (PXR) or constitutive androstane receptor (CAR) and lead to pharmacokinetic herb-drug interactions. The aim of the study was to determine the influence of valerian on the expression level of CYP3A1 (homologue to human CYP3A4) as well as nuclear receptors PXR, CAR, RXR, GR, and HNF-4*α*. Male Wistar rats were given standardized valerian extract (300 mg/kg/day, p.o.) for 3 and 10 days. The expression in liver tissue was analyzed by using real-time PCR. Our result showed a decrease of CYP3A1 expression level by 35% (*P* = 0.248) and 37% (*P* < 0.001), respectively. Moreover, *Valeriana* exhibited statistically significant reduction in RXR (approximately 28%) only after 3-day treatment. We also demonstrated a decrease in the amount HNF-4*α* by 22% (*P* = 0.005) and 32% (*P* = 0.012), respectively. In case of CAR, the increase of expression level by 46% (*P* = 0.023) was noted. These findings suggest that *Valeriana officinalis* extract can decrease the CYP3A4 expression and therefore may lead to interactions with synthetic drugs metabolized by this enzyme.

## 1. Introduction

*Valeriana officinalis* (valerian) is one of the most popular medicinal plants in Europe and the United States. It is mainly used to treat insomnia, mild anxiety and reduce muscle tension [1, 2]. Valerian root contains a complex mixture of chemical compounds, especially valerenic acid and its derivatives that may act synergistically to exert sedative effects [3, 4]. Many experimental researches have reported that the use of valerian preparations with conventional medications can lead to the potential herb-drug interactions. Based on presented studies, it is claimed that valepotriates may prolong the action of barbiturates whereas intraperitoneal administration of valerenic acid may cause an increase of the anesthetic effect of pentobarbital [5, 6]. Despite the widespread use of valerian preparations, the data regarding the safety as well as herb-drug interactions are very limited. Hence, it is suggested that the phytochemical profile of* Valeriana officinalis* may have influence on the activity of cytochrome P450 (CYP) enzymes involved in phase I metabolism of drugs and other xenobiotics.

The CYP2C, CYP2D, and CYP3A subfamilies are the most active CYPs responsible for drug biotransformation, especially the CYP3A4 isoform that is involved in the metabolism of more than 50% of clinically used drugs [7, 8]. CYP3A4 enzyme constitutes about 30–40% of total hepatic CYP content and it is also present in small intestine. The expression of CYP3A4 is regulated by a number of chemical inducers and other factors trough nuclear receptor signaling pathways involving mainly pregnane X receptor (PXR) and constitutive androstane receptor (CAR) [9, 10]. The mechanism of action involves binding with retinoid receptor (RXR) to form a heterodimer which has high DNA binding affinity [11]. On the other hand, it seems that glucocorticoid receptor (GR) mediates the induction of CYP3A4 transcription in human hepatocytes after dexamethasone administration [12]. Moreover, other studies have shown that the HNF-4*α* has the ability of binding to the CYP3A4 promoter [13]. In addition, the above-mentioned factors such as PXR, CAR play an important role in the transcriptional regulation of other genes responsible for the transport and metabolism of drugs including MDR1, SULT, and UGT [14, 15]. Similar to CYP3A4, PXR is mainly expressed in liver and small intestine [16, 17]. It is suggested that activation of PXR by ligand is one of the principal mechanisms underlying the induction of CYP enzymes that may lead to interactions of clinically used drugs [18]. Ligands of PXR are a variety of endogenous substances (steroid hormones and steroid metabolites) and dietary compounds (coumestrol, carotenoids) as well as pharmaceuticals (rifampicyna, phenobarbital, and nifedipine) [9, 19]. Moreover, it was shown that hyperforin of St. John's wort is the most potent PXR activator and can induce the CYP3A4 expression leading to an undesirable pharmacological effect of clinically used drugs [20]. The nuclear receptor CAR as a xenobiotic responsive transcription factor is mainly expressed in liver and it mediates the induction of CYP2B6 and also CYP3A4 [21]. Phenobarbital is a classic CAR-mediated inducer of xenobiotic metabolism, whereas deactivators and inverse agonists, such as androstanol and clotrimazole, deactivate the above nuclear receptor [11, 16]. Moreover, CAR may act differently in comparison to the traditional receptors because it can be constitutively active without ligand. However, this mechanism of CAR regulation is not totally understood.

Many herbal medicines that are often used in prevention of civilization diseases as well as an additive comedication during therapy with synthetic drugs can have influence on the activation of nuclear receptors and CYP enzymes leading to herb-drug interactions and the appearance of undesirable side effects. Therefore, the studies concerning molecular mechanism of interactions between herbal and synthetic drugs are very important for the safety evaluation and efficacy of pharmacotherapy. Hence, the aim of our study was to determine the influence of valerian extract on the expression level of CYP3A1 (homologue to human CYP3A4) enzyme as well as PXR, CAR, RXR, and GR receptors responsible for transcriptional regulation of this isoform. Furthermore, we present the first report on the impact of valerian on HNF-4*α* associated with constitutive expression of several CYP enzymes in the liver.

## 2. Material and Methods

### 2.1. *Valeriana officinalis* Extract

The standardized extract of* Valeriana officinalis* used in this study was obtained from Phytopharm Kleka S.A. (Poland). The chemical composition of the essential oil of* Valeriana officinalis* was determined by using gas chromatography-mass spectrometric GC/MS techniques according to methods described by Adams [22] and Raal et al. [23] in the Department of Quality Control of Medicinal Products and Dietary Supplements at the Institute of Natural Fibres and Medicinal Plants (Poznan, Poland). The basic constituents of the essential oil were isovaleric acid (2.1%), bornyl acetate (8.6%), myrtenyl acetate (3.9%), myrtenyl isovalerate (2.4%), *α*-pinene (0.4%), *α*-fenchene (0.9%), camphene (0.8%), valerianol (1.6%), valeranone (2.5%), valerenal (5.2%), and sesquiterpene alcohol (4.5%). The content of valerenic acid (0.82%) was determined by using high performance liquid chromatography (HPLC) according to European Pharmacopoeia 6.0.

### 2.2. Chemicals

Ketoconazole was purchased from Sigma-Aldrich Chemical Company (St. Louis, MO, USA). Dexamethasone was purchased from PGF Cefarm-Poznan Sp. z o.o. (Poznan, Poland).

### 2.3. Animals and Treatment

The experiment with male Wistar rats (200–250) was performed in accordance with Polish governmental regulations (Decree on Animal Protection Dz. U. 97. 111. 724, 1997) and in an agreement with Local Ethic Committee of the Use Laboratory Animals in Poznan (number 43/2005). Male Wistar rats were housed in plastic cages at the Department of Pharmacology, Poznan University of Medical Sciences. Animals were kept in a climate-controlled room with 12 h light/dark cycle and allowed access to a commercial rat chow and tap water* ad libitum*. They were acclimatized for at least a few days prior to experiment. All rats were randomly divided into 8 groups from A to H (ten per group). Group A was treated with the standardized extract of* Valeriana officinalis* 300 mg/kg/day p.o. for 3 days, whereas group B was treated with the same extract like group A 300 mg/kg/day p.o. but for 10 days. Two groups C and D were given dexamethasone (50 mg/kg/day i.p.) as a potent CYP3A1 inducer for 3 and 10 days, respectively. The next two groups were treated with ketoconazole (10 mg/kg/day, i.p. for 3 and 10 days) as a potential inhibitor of CYP3A1 enzyme. Groups G and H used as controls were fed standard diet. Sixteen hours after the last administration, rats were decapitated. The liver tissues were immediately frozen in liquid nitrogen and stored at −80°C.

### 2.4. Quantitative Real-Time PCR

**Figure 1:**
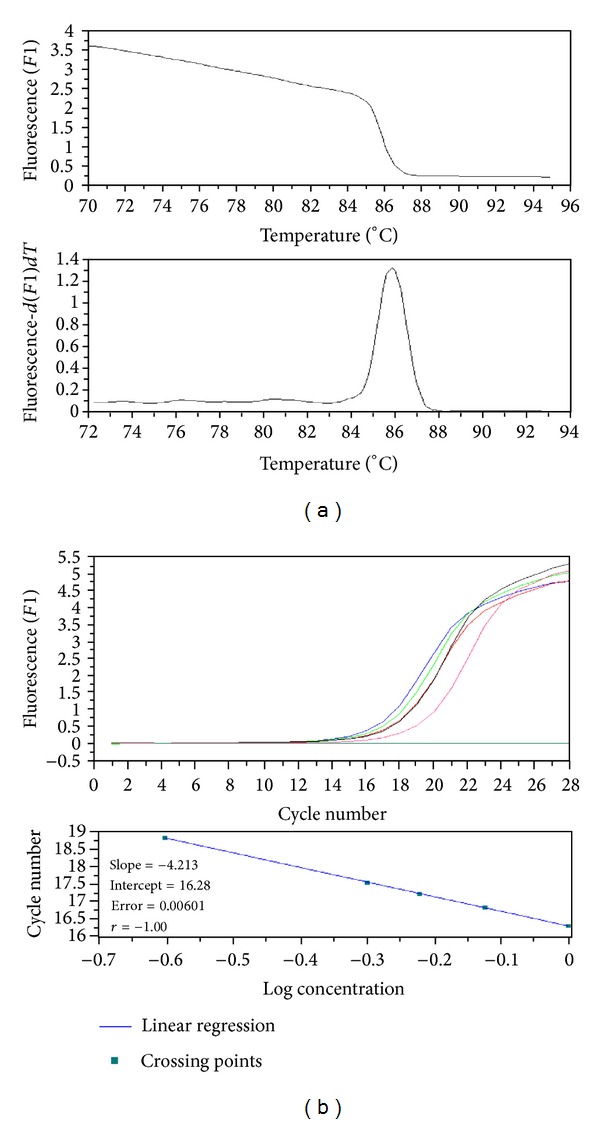
Real-time PCR reaction for analyzed gene. (a) Melting curve analysis, Tm = 85.90°C; (b) standard curve.

To quantitate the expression level of target genes, cDNA was synthesized from total RNA isolated from liver using the SuperScript III First-Strand Synthesis System (Invitrogen) and oligo (dT)_20_ primer according to the manufacturer's protocol. Real-time PCR (RT-PCR) was carried out using a LightCycler TM Instrument (Roche, Germany) and a LightCycler Fast Start DNA Master SYBR Green I kit (Roche, Germany). Amplicon size and reaction specificity were confirmed by agarose gel electrophoresis and melting curve analysis. The primers and PCR conditions used for CYP3A1 and GAPDH amplifications were described by Mrozikiewicz et al. [24]. The sequences of the primers for PXR, CAR, RXR, GR, HNF-1*α*, and HNF-1*α* designed with the use of the Oligo 4.0 program (National Biosciences, USA) were as follows: PXR-F: 5′-TCC ACT GCA TGC TGA AGA AG-3′; PXR-R: 5′-AAC CTG TGT GCA GGA TAG GG-3′; CAR-F: 5′-GGA GGA CCA GAT CTC CCT T-3′; CAR-R: 5′-GAC CGC ATC TTC CAT CTT GT-3′; RXR-F: 5-CCT GAG TTC TCC CAT CAA TG; RXR-R: 5-GAC GCC ATT GAG GCC TAG A; GR-F: 5-CTG GAA TAG GTG CCA AGG CT; GR-R: 5-CCG TAA TGA CAT CCT GAA GCT; HNF4*α*-F: 5-CTG GAG GAT TAC ATC AAC GAC; HNF4*α*-R: 5-GTG TTC TTG CAT CAG GTG AG. The thermal cycling conditions for nuclear receptor were as follows: for PXR, 35 cycles of 95°C for 8 s, 55°C for 8 s, and 72°C for 8 s; for CAR, 35 cycles of 95°C for 8 s, 58°C for 8 s, and 72°C for 8 s; for RXR, 35 cycles of 95°C for 8 s, 57°C for 7 s, and 72°C for 8 s; for GR, 40 cycles of 95°C for 10 s, 58°C for 8 s, and 72°C for 8 s; for RXR, 35 cycles of 95°C for 8 s, 57°C for 7 s, and 72°C for 8 s; for HNF-4*α*, 40 cycles of 95°C for 8 s, 57°C for 7 s, and 72°C for 8 s. Standard curves were prepared from dilution of cDNA and generated from a minimum of four data points (Figure 1). The data were evaluated with the Roche LightCycler Run 5.32 software. GAPDH was used as a housekeeping gene for normalization (endogenous internal standard).

### 2.5. Statistical Analysis

The results were expressed as mean ± SEM. All data were analyzed using the SPSS 17.0 for Windows program by one-way ANOVA test. The values of *P* < 0.05 were considered as significance.

## 3. Results

**Figure 2:**
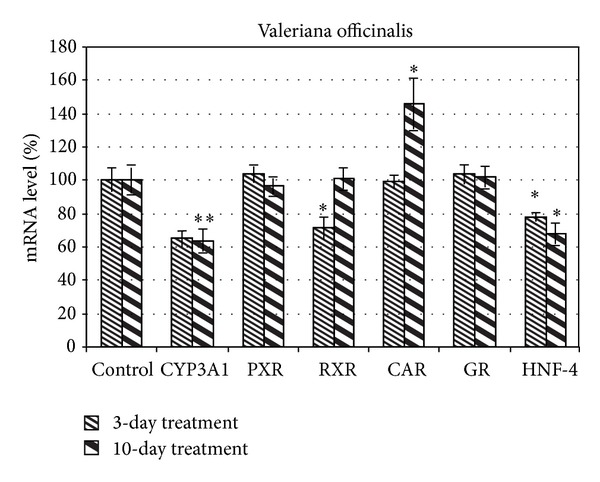
The influence of* Valeriana officinalis* on the expression level of CYP3A1 and nuclear receptors in the rat liver tissues of male rats after 3 and 10 days of administration. The control groups were defined as 100%. Data were presented as mean ± SEM of 10 rats in each group. *P < 0.05 as compared with the control group (one-way ANOVA test). **P < 0.001.

In this study, we investigated the influence of standardized extract of* Valeriana officinalis* on mRNA abundance of CYP3A1 and PXR, CAR, RXR, and GR receptors as well as HNF-4*α* (Table 1). The level of mRNA expression in liver tissues was analyzed by using RT-PCR method. Our study showed that administration of valerian extract caused a decrease of CYP3A1 expression level by 35% (*P* = 0.248) and 37% (*P* < 0.001), respectively (Figure 2). Moreover,* Valeriana* exhibited statistically significant reduction in RXR by more than 28% (*P* = 0.034) only after 3-day treatment. We also observed a decrease in the amount of HNF-4*α* by 22% (*P* = 0.005) and 32% (*P* = 0.012) after 3 and 10 days, respectively. In case of receptor CAR, the increase of mRNA level by 46% (*P* = 0.023) after 10 days was noted. No significant changes in PXR and GR mRNA level after extract administration were observed.

**Figure 3:**
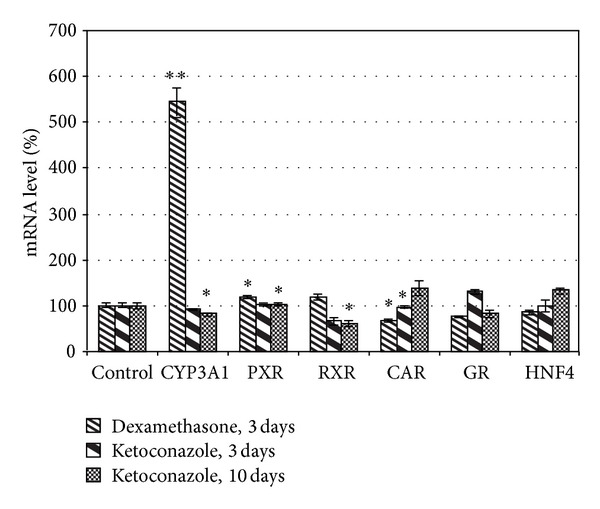
Effect of dexamethasone (DEX) and ketoconazole (KET) on the expression level of CYP3A1 and nuclear receptors in the rat liver tissues after 3 and 10 days of administration. The control groups were defined as 100%. Data were presented as mean ± SEM (*n* = 10). *P < 0.05 was considered as significance (one-way ANOVA test). **P < 0.001.

Additionally, we also used synthetic substances as model substances for the rat CYP3A1 enzyme. Our results showed that dexamethasone as a potential inducer of CYP3A1 produced significant increase of its expression level in the liver tissues by over 4.4-fold (*P* < 0.01) after 3 days whereas long-term administration of this substance caused death of rats after 4 days as a result of hepatotoxicity effect (Figure 3). Analyzing PXR and RXR mRNA profile after dexamethasone administration, there was observed a small increase of their expression levels by 19% (*P* = 0.033) and 18% (*P* = 0.302) versus control group. In contrast to CYP3A1, the levels of CAR and GR mRNA were reduced by 31% (*P* = 0.16) and about 22% (*P* = 0.008), respectively. HNF-4a level decreased slightly by almost 15% after dexamethasone.

In case of ketoconazole administration as an inhibitor for CYP3A1, it was shown a small decrease of CYP3A1 mRNA level by 7% (*P* = 0.3) and 18% (*P* = 0.013), respectively. At the same time, a reduction in RXR by over 33% (*P* = 0.061) and 39% (*P* = 0.016) was reported. No statistically significant differences were observed for PXR both after 3 and after 10 days. However, the inductive effect of ketoconazole was observed for nuclear receptor CAR by 38% (*P* = 0.044) and HNF-4*α* by 33.5% (*P* = 0.047) after 10 days. Moreover, the significant induction of hepatic GR fraction by over 31% (*P* = 0.002) after the short-term use of valerian was obtained.

## 4. Discussion

*Valeriana officinalis* is commonly used as a traditional medicinal product. Despite its widespread use, data concerning the molecular mechanism and consequences of herb-drug interactions is poorly known and needs further explorations. In addition, there is little information about the influence of valerian on the CYP3A4 expression level and potential interactions with clinically used drugs by modulation of its activity. It is necessary to underline that so far there were only the analyzed influence of* Hypericum perforatum* and* Glycine max* on the expression level of PXR as well as the effect of* Ginkgo biloba* on the activity of PXR and CAR. Currently, there are no treatments regarding the influence of* Valeriana officinalis* on mRNA level of the above nuclear receptors.

Therefore, in our study we determined the influence of a standardized extract of* Valeriana officinalis *on the expression level of CYP3A1 (homologue to human CYP3A4) as well as PXR, CAR, RXR, GR, and HNF-4*α* recognized as key factors in transcriptional regulation of this enzyme. Based on these findings, we showed that valerian extract significantly reduced the CYP3A1 mRNA level. Hence, our results suggest that standardized herbal extract can decrease the CYP3A4 activity* in vivo* and may participate in clinically significant interactions with drugs metabolized by this enzyme, since the inhibition of the CYP3A4 enzyme causes a decrease of drug metabolism leading to an undesirable pharmacological effect and the appearance of toxic symptoms of overdose.

Similar effect to our experiment was observed by Budzinski et al. [25]. They showed that both ethanolic extract of* Valeriana officinalis* and valerenic acid inhibited the CYP3A4 activities in human liver microsomes. Other studies have also reported that extract from the valerian root reduced CYP3A4 activity* in vitro* [6, 26]. The results of Lefebvre et al. [6] exhibited moderate-to-high (35–88%) inhibition of the enzyme activity after administration of valerian root products. However, it is difficult to state how much CYP3A4 inhibition might occur* in vivo* during application of commercial valerian extracts that have different chemical profile. Besides, in another study, it was also noted that organic extract of the root caused a higher decrease of CYP3A4 activity than individual components such as valerenic acid [26, 27]. Hence, it is suggested that the isolated substance from the valerian root may act otherwise in comparison to the whole extract.

In contrast to our study, according to Donovan et al. [28], the valerian administration (1000 mg/day) for 14 days in the twelfth healthy volunteers caused no significant changes in CYP3A4 and CYP2D6 activity. They suggested that* Valeriana officinalis* does not participate in clinically significant interactions with conventional drugs metabolized by these enzymes. Moreover, Gurley et al. [29] obtained similar results using 375 mg/day of valerian for 28 days in the twelfth healthy volunteers. Therefore, it is claimed that the above findings of* in vivo* and* in vitro *investigations regarding the influence of valerian on CYP3A4 enzyme activity are discordant. It may result from a small number of volunteers participating in these experiments, different phytochemical composition used preparations and consequently from period of their chronic administration and dosage.

In our experiment, we used a higher dosage of valerian in comparison to human in order to determine the potential influence of this extract on the CYP3A4 expression level with reference to its nuclear receptors and to asses a risk of the appearance of interactions with synthetic drugs metabolized by this enzyme. We tried to define the relationship between CYP3A expression and selected transcription factors level. Our results showed that valerian extract may cause differential expression profile of PXR, GR, and HNF-4*α* dependent on the time of administration. Hence, it is difficult to explain the molecular mechanism of CYP3A4 regulation by nuclear receptor PXR because some findings suggest that CYP3A4 expression can be regulated indirectly by PXR. The results of our study indicate that PXR and RXR level may be associated with transcriptional activation of CYP3A1. We noted a slight increase in transcript levels of PXR and RXR factors after dexamethasone. On the other hand, only RXR was reduced by valerian extract in the short-term use. This implies that the mechanism of inhibitory action of the active ingredients from valerian on CYP3A is not associated with GR and PXR transcription. Moreover, in case of nuclear receptor CAR, our findings showed certain correlation with the expression level of CYP3A1 after model substances administration. Similar effect was observed for standardized extract of* Valeriana officinalis* containing 0.82% valerenic acid. However, for confirmation of the presented dependence concerning expression between CYP3A4 and CAR, it needs further explorations in order to fully explain the molecular mechanism of CYP3A4 regulation by this receptor. Furthermore, it is necessary to underline that dexamethasone as a potent inducer of CYP3A can activate the glucocorticoid receptor (GR) leading to the induction of nuclear receptor PXR and to the increase of CYP3A4 expression by endogenous and exogenous compounds [30]. In our study, we observed a significant decrease in CAR, GR, and HNF-4*α* mRNA. This is the first study to elucidate the effect of valerian on the investigated transcription factors that modulate the activity of CYP3A. It is desirable to carry out further extensive research: in particular, the extract may have modulating properties to other cytochrome P450 enzymes.

## 5. Conclusions

In summary, we have described the influence of standardized* Valeriana officinalis* extract on the CYP3A1 (homologue to human CYP3A4) gene expression and PXR, RXR, CAR, GR, and HNF-4*α* responsible for transcriptional regulation of this enzyme. Our findings showed that* Valeriana officinalis* extract decreases the CYP3A1 expression and may lead to interactions with synthetic drugs metabolized by human CYP3A4 enzyme. It can be assumed that human CYP3A4 expression can be regulated by RXR receptor after valerian administration. It is also suggested that long-term reduction of CYP3A is associated with a decrease of HNF-4a. Hence, it is necessary to underline that further* in vivo* studies regarding the potential influence of herbal extracts on CYP enzymes activities and their transcription factors might be helpful for the safety evaluation and efficacy of pharmacotherapy.

## Figures and Tables

**Table 1:** The effect of synthetic substances (DEX, KET) and *Valeriana officinalis* on expression level of analyzed genes in male rats (*n* = 10).

Substance	(mean% ± SEM%) 3 days	P*	(mean% ± SEM%) 10 days	P*
CYP3A1				
DEX (50 mg/kg)	544.36 ± 34.07	**<0.001**	**—** ^ #^	**—** ^ #^
KET (10 mg/kg)	92.92 ± 4.07	0.3	81.97 ± 5.56	**0.013**
VAL (300 mg/kg)	65.37 ± 6.35	0.248	63.36 ± 6.66	**<0.001**
PXR				
DEX (50 mg/kg)	118.72 ± 5.49	**0.033**	**—** ^ #^	**—** ^ #^
KET (10 mg/kg)	101.27 ± 4.31	0.879	105.52 ± 6.7	0.686
VAL (300 mg/kg)	103.66 ± 5.61	0.62	96.35 ± 5.77	0.62
CAR				
DEX (50 mg/kg)	68.85 ± 4.74	0.16	**—** ^ #^	**—** ^ #^
KET (10 mg/kg)	97.14 ± 6.36	0.72	138.44 ± 17.71	**0.044**
VAL (300 mg/kg)	99.0 ± 4.94	0.837	146 ± 15.73	**0.023**
RXR				
DEX (50 mg/kg)	117.79 ± 9.67	0.302	**—** ^ #^	**—** ^ #^
KET (10 mg/kg)	67.34 ± 8.4	0.061	60.63 ± 9.48	**0.016**
VAL (300 mg/kg)	71.39 ± 7.72	**0.034**	101.19 ± 7.27	0.917
GR				
DEX (50 mg/kg)	77.54 ± 4.05	**0.008**	**—** ^ #^	**—** ^ #^
KET (10 mg/kg)	131.66 ± 5.68	**0.002**	82.88 ± 10.04	0.145
VAL (300 mg/kg)	103.63 ± 6.78	0.644	101.70 ± 7.21	0.859
HNF-4*α*				
DEX (50 mg/kg)	85.13 ± 9.46	0.196	**—** ^ #^	**—** ^ #^
KET (10 mg/kg)	99.77 ± 15.62	0.987	133.51 ± 7.65	**0.047**
VAL (300 mg/kg)	77.84 ± 3.92	**0.005**	67.94 ± 7.37	**0.012**

The control groups fed standard diet were defined as 100%. *P < 0.05 as compared with the control group (one-way ANOVA test). DEX: Dexamethasone; KET: ketoconazole; VAL: *Valeriana officinalis*. ^#^No data.
